# On the Clinical Study of the Heart-Sounds

**Published:** 1858-10

**Authors:** Austin Flint

**Affiliations:** Prof. of Clinical Medicine and Auscultation, in the N. O. School of Medicine


### SELECTIONS.

From the N. O. Med. News and Hospital Gazette.

*On the Clinical Study of the Heart-Sounds.* By Austin Flint, M. D., Prof. of Clinical Medicine and Auscultation, in the N. O. School of Medicine.

LETTER NO. I.

Prof. Fenner—*Dear Sir:* In compliance with your request, I will endeavor to give in this and a succeeding letter, prepared for publication in the *Medical News and Hospital Gazette*, a *résumé* of the results of the study of the heart-sounds in health and disease—a subject on which I have of late bestowed some attention. The modern history of auscultation is to be dated from the epoch of the application to the chest of a quire of paper rolled into a clyinder, by Laennec in the case of a patient affected with disease of the heart. But the illustrious founder of physical exploration at once directed his researches to the phenomena pertaining to the respiratory system, and, although he was the first to observe adventitious sounds or *murmurs*, in certain cardiac affections, he accomplished, in behalf of the diagnosis of the latter, little in comparison with the results of his investigation of pulmonary diseases. This was in a great measure owing to his holding false notions respecting the production of the normal sounds of the heart. The correction of these errors by means of the investigations of Hope and others, prepared the way for a better knowledge of the physical signs referable to this organ. The importance of an accurate acquaintance with the heart-sounds in health, as regards their sources, their relations to the cardiac movements, and the mechanism of their production, is obvious to any one who has given attention to the subject. But the importance of the *clinical* study of these sounds, with reference to these points, has not been sufficiently appreciated. A dependance too exclusive has been placed on experiments by means of vivisections and on the few instances of ectopia which have fallen under observation. From these experiments valuable results have been obtained, more particularly as regards the movements of the heart, but also, to some extent, with respect to the normal sounds. There is still scope for experimental researches with the heart exposed to view. But it must be apparent, on a little reflection, that, so far as auscultation is concerned in these experiments, it involves difficulties and incidental circumstances which render it, in a measure at least, unsatisfactory and unreliable. Suppose that in an animal of large size we have succeeded in opening the chest and pericardium, so that the heart may be seen and handled for a considerable period, its movements being maintained by artificial respiration, one important normal condition is necessarily wanting, viz: contact with the thoracic parieties; nor have we a right to assume that, after such an operation, the conditions as respects the quantity of blood within the cavities, the contractions of the walls and the flap of the valves, are such as to render the sounds faithful types of those incident to the normal state. But, irrespective of these considerations, auscultation must be practiced applying the stethoscope immediately upon the heart, a muscular organ in almost unceasing activity. How can the sounds as thus heard, represent fairly those obtained by auscultation with the stethoscope placed upon the chest? These remarks will, of course, equally apply to cases of ectopia. A single trial suffices to show that the study of the heart sounds thus pursued, is unsatisfactory, and to a certain extent, unreliable. On the other hand, the *clinical* study of the normal sounds (and by this expression I mean the study by means of examination of the healthy chest,) is capable of yielding results, reliable and satisfactory, which lead to important conclusions concerning the origin of these sounds and the agencies involed in their production. If to the clinical study in health, be conjoined the clinical study in disease, together with the information respecting the movements of the heart obtained by vivisections, our knowledge of the normal heart-sound may be rendered sufficiently complete for all practical purposes —that is, so far as this knowledge is necessary to serve as a point of departure for obtaining, by means of clinical observation, an acquaintance with the abnormal modifications of the sounds of the heart, and the signficance which belongs to the latter as physical signs of cardiac affections.

The importance of the abnormal modfications of the heart-sounds, as constituting physical signs of disease, have hitherto been inadequately estimated. Of the small value attached to them in this point of view, the trifling space generally allotted to their consideration in works treating of diseases of the heart, is sufficient evidence. A

few sentences, or at most, a few pages only, are devoted to this subject. The attention of auscultators appears to have been absorbed by the study of adventitious sounds, or murmurs.* I am far from wishing to depreciate the value which the latter possess in diagnosis. In determining the existence, seat, and some of the effects of valvular lesions, they are invaluable. They by no means, however, meet all the wants of the diagnostician. Their presence, as is well known, is not, in itself, evidence of organic disease of the heart. The practitioner is to distinguish between organic and inorganic murmurs.— This, it will be granted, may be done without much difficulty in the great majority of cases. Assuming that this is done, and excluding the rare instances in which a bellows sounds accompanies uncomplicated enlargement of the heart, what information do murmurs afford beyond the fact of valvular lesions and their situation? Do they indicate the extent of damage which the lesions have occasioned—in other words, the amount of obstruction or regurgitation resulting therefrom? No one will venture to answer affirmatively the latter question. The intensity and quality of murmurs have no special pathological significance. A rough or intense murmur may be due to lesions which are insignificant, involving no immediate danger; or, on the other hand, the most serious lesions may be accompanied by a soft feeble murmur. To be able to determine the injury which existing lesions have occasioned, is of vastly more importance than to ascertain the single fact that lesions exist, and to localize them; for valvular lesions give rise to danger and inconvenience only in proportion as they render the valves insufficient or induce contraction of the orifices. It were easy to adduce cases in illustration of the correctness of these statements, which will be admitted by all who have given attention, practically to diseases of the heart. Now, the study of the abnormal modifications of the heart-sounds, taken in connection with the murmurs, is capable of supplying, in a great measure, that which the study of the latter alone is incapable of affording, viz: information respecting the extent of valvular lesions, and the damage which they have occasioned. Attention to the heart-sounds in disease, moreover, affords aid, in certain cases, in the localization of these lesions.

In the clinical study of the heart-sounds in health and disease, the two sounds are, of course, to be made separate subjects of investiga-

* It is hardly necessary to remind the reader that, conventionally, the term *heart-sounds* is now restricted to the normal sounds and their abnormal modifications; while all adventitious sounds are included under the name of *murmurs*.

tion. It is quite needless to premise that these sounds are distinguished, respectively, as the *first* or *systolic*, and the *second* or *diastolic* sound; that the *first* sound takes place synchronously with the apex-beat and the pulsation of the large arteries near the heart, while the *second* sound occurs after the systolic movement of the ventricles; the two sounds succeed each other in a certain rythmical order, and that, combined and forming what is called the beat or revolution of the heart, they follow after successive intervals nearly or quite uniform; and, finally, that each sound and interval has a certain relative duration, the sounds differing from each other in pitch, quality and intensity, as well as in length. Proceeding to present, as concisely as possible, those points pertaining to each of the sounds, which are of practical importance with reference to diagnosis, I shall commence with the second sound. It might seem more natural to give precedence to the first sound. The reason for reversing the natural order is, that the second is the simpler sound; as regards its seat and mechanism it is better understood, and its consideration will prepare for the better study of the first sound. The remainder of this letter will be devoted to the second or diastolic sound in health and disease, and in a subsequent letter the first or systolic sound will be considered.

SECOND OR DIASTOLIC SOUND OF THE HEART IN HEALTH AND DISEASE.

That this sound is produced mainly at the orifices of the aorta and pulmonic artery, and that the semilunar valves are essential to its production, may be assumed as sufficiently established. The commonly received explanation is, that the sound is due to the sudden, forcible expansion of these valves, caused by the recoil of the arterial coats succeeding the dilatation by the column of blood propelled from the ventricles. The force with which these valves are unfolded and rendered tense, and the consequent intensity of sonorous vibrations, will obviously, other things being equal, be proportionate to the quantity of blood propelled though the aorta and pulmonic artery by the ventricular systole and the muscular power of the ventricles. The clinical study of this sound in health confirms the correctness of this localization. The sound has its maximum of intensity above the base of the heart, at the points where the aorta and pulmonary artery approach nearest to the thoracic walls, viz: in the second intercostal spaces on either side of the chest, near the sternum.

This sound emanates from the aortic and pulmonic orifices. Now, is it true that the aortic and the pulmonic sound may be disconnected and distinguished from each other in certain situations, as has been of late maintained by some observers? Plainly, this is a question to be settled by comparing the sound heard at different points on the chest in a series of healthy persons. With reference to this and other points relating to the normal heart-sounds, I examined with care twenty-five healthy persons, selected for this purpose, noting the results at the moment of the examination, and afterwards subjecting them to analysis. In nearly all of these persons, the sound in the second intercostal space, near the sternum, presented, on the two sides of the chest, well marked differences in pitch, duration, intensity, and apparent proximity to the ear. On the left side it is, relatively, more feeble, dull, distant and longer. On the right side it is more intense, more acute, nearer and shorter. These differences can hardly be due to modifications received by the sound in its transmission to the ear; and, hence, the point to a distinct source of the sound which, in health, predominates in each of these situations. Taking into view the anatomical disposition of the aorta and pulmonic artery, we arrive at the conclusion, that the sound in the second intercostal space, on the right side, emanates from the aorta, and that on the left side, from the pulmonic artery. Observation in cases of disease confirms the correctness of this conclusion. When the aortic valves are the seat of lesions which prevent their play, the second sound on the right side is impaired or wanting; that on the left side remaining, and preserving its normal characters.*

This point settled, another inquiry arises: The second, as heard in the other situations within and near the præcordia—at what points is it aortic, i. e., emanating from the aortic orifice, and at what points is it pulmonic, i. e., referable to the orifice of the pulmonary artery?—This question, plainly, is to be settled by auscultation of the sound in different situations, and comparing its characters succesively with the aortic and the pulmonic sound as heard in the second intercostal space on the two sides, near the sternum. An analysis of the results obtained by examining twenty-five healthy persons, leads to the following conclusions: The sound presents the characters which distinguish that emanating from the pulmonic orifice, in a few instances, in the third intercostal space on the left side, and over the body of the heart with-

* "Ce que nous ne pouvons produire traumatiquement et brusquement, la nature le fait sans nous, d'une maniere graduelle et sans porter de profondes perturbations dans les fonctions de l'organe, comme ferait une vivisection: en sorte que nous considerons ces lesions pathologiques comme de tres-bonnes *experiences* cliniques."—*Bouillaud-Lecons cliniques sur les maladies du cœur*, 1853.

in the 'superficial cardiac region.' In a large proportion of instances it presents these characters over the inferior boundary of the heart, just above the xiphoid cartilage. In other situations within and near the præcordia, in which the second is heard, it uniformly presents the characters distinctive of that emanating from the aortic orifice.

Other important conclusions are deduced from the results of the clinical study of the second sound in health. The sound has a strongly marked quality which conveys the idea of valvular action, and may be distinguised as a valvular quality. It preserves this valvular quality wherever it is heard. It does not present any marked variations in different situations, save, as regards intensity. It is characterized by uniformity, and is evidently an unmixed sound, that is due exclusively to a single element, in these respects differing, notably, from the first sound of the heart. As regards intensity, it varies far less than the first sound, being affected in a less degree by the causes which increase or diminish the force of the heart's action. In the rythmical succession of the two sounds at the base of the heart, the stress appears to fall on the second; the latter is said to be accentuated. Over the body and apex, it is otherwise; the accentuation is on the first sound. But without the præcordia, at points more or less removed from the heart, when the two sounds are heard, the second sound is accentuated; showing that, although within the limits of the præcordial region the intensity of the first sound preponderates, the second sound is more widely diffused. This is also shown by the fact that in carrying the stethoscope away from the heart, the first sound, as a rule in health, becomes inappreciable sooner than the second. The explanation of these results of observation will appear when the differerent elements composing the first sound are considered.

Directing attention now to the study of the second sound in disease, we are at once brought to the practical importance of distinguishing between the aortic and the pulmonic sound. In certain cases of disease, the sound referable to the pulmonic artery becomes mere intense than that referable to the aorta. Prof. Skoda, of Vienna, was I believe, the first to call attention to the diagnostic significance of this intensification or reinforcement of the pulmonic second sound. It is at first view mysterious that there should exist a relation of sequence between this event and lesions of the mitral orifice producing either obstruction or regurgitation, or both; but well known pathological laws render it intelligible. The first effects of mitral contraction or insufficiency, are distension and dilation of the left auricle; incidental to these effects is pulmonary congestion, and the third link in the chain of sequences is hypertrophy of the right ventricle. The increased power of the right ventricle due to its hypertrophy, and the resistance to the current of blood in its circuit through the lungs in consequence of the congestion of the pulmonary vessels, increase the distension of the pulmonic artery by the column of blood propelled by the ventricular systole; hence, a corresponding increase of the recoil of the coats of the artery after the systole, and an abnormal intensity of the second sound emanating from this artery. Intensification of the pulmonic second sound is thus a sign of hypertrophy of the right ventricle, a pathological effect, in the vast majority of cases of mitral obstruction or regurgitation, either separately or combined. I have given the explanation of the fact, but the first questions is, does clinical observation afford sufficient evidence of the fact? I can testify from considerable experience to the intensification of the pulmonic sound in the great majority of the cases of mitral lesions which have eventuated in hypertrophy of the right ventricles. The greater intensity of this sound, however, as compared with the aortic, is to be explained, in a measure, by the diminished intensity of the aortic sound as an effect of the reduced quantity of blood propelled through the aorta in cases of mitral contraction and of insufficiency. This will account, in part, for the greater relative intensity of the pulmonic sound. But, aside from this explanation, a positive augmentation of the intensity of the latter undoubtedly takes place in a large proportion of cases. The sign is valuable, taken in connection with the evidence afforded by other signs of the existence of mitral lesions, as denoting that the heart has begun to suffer from the effects of these lesions, which, until they lead to enlargement, are not attended by much danger or inconvenience. Prof. Skoda does not appear to attach sufficient importance to the great relative intensity of the pulmonic sound being measurably due to the weakening of the aortic sound. The positive augmentation only, of course, is evidence of hypertrophy of the right ventricle. It is important, therefore, to decide, individual cases, whether the aortic sound preserves its normal intensity or not. This can generally be done by observing, not only its absolute intensity in the right second intercostal space, but its intensity in situation removed from the præcordia, where it is usually found in health to predominate over the first sound. It is also to be borne in mind that morbid conditions pertaining to the lungs occasion greater intensity of the second sound in the second intercostal space on the left side. A tuberculous deposit near the summit of the left lung has this effect; so, chronic pleurisy affecting the left side after absorption of the effused liquid, etc.

Attention to the aortic second sound in connection with the diagnosis of lesions affecting the valves and orifice of the aorta, is a practical point of much importance. The existence of lesions in this situation is shown by the presence of aortic murmurs. But the characters of these murmurs furnish no evidence of the amount of damage to the aortic valves which the lesions have occasioned. How are we to judge of the condition of these valves prior to that stage of disease when enlargement of the left ventricle is proof that aortic obstruction or regurgitation, or both, have existed for a considerable period? So long as the aortic second sound retains its normal intensity, notwithstanding the presence of aortic murmur, the valves are not greatly damaged. A loud murmur may be due to aortic lesions which have left the valves intact, and are thefore of comparatively little consequence. This statement is applicable to a diastolic, as well as to a systolic aortic murmur. The aortic valvular sound thus becomes a highly important criterion of the condition of the valves, in connection with aortic murmurs. Immobility of the aortic valves involves suppression of the aortic second sound, the pulmonic sound remaining. Repeated illustrations of this fact have fallen under my observation. Abnormal weakness of the aortic sound will correspond to the dregree of contraction, rigidity or destruction of the valves, which the lesicns have occasioned. In making the weakness of the sound the criterion of the amount of damage which the valves have sustained, it is important to bear in mind that this result also occurs as a consequence of mitral obstruction and regurgitation. As evidence of the impaired condition of the aortic valves, its value is therefore lessened in the cases in which, as not unfrequently happens, mitral and aortic lesions co-exist The muscular power of the heart is also to be taken in account. Feebleness of the contractions of the left ventricle, of course involves a feeble aortic sound. But as the causes inducing diminished muscular power in general act on the whole heart, the pulmonic and the aortic sound are alike expelled. Both, however, are affected under these circumstances, far less than the first sound of the heart.

In another point of view, attention to the aortic second sound is of practical importance in diagnosis, viz., in the localization of a diastolic murmur. A diastolic murmur proceeds either from a retrograde aortic current, or from the direct current through the mitral orfice. The occurrence of a mitral diastolic murmur, except in very rare instances, is ignored by some writers; but I am satisfied that I have repeatedly met with instances in which it was present. The discrimination of this from an aortic regurgitant murmur, is attended with difficulty.—The aortic murmur is said to be post-systolic and the mitral murmurpre-systolic; but when it is considered that these distinctions of time are to be made within less than one third of a second, it is plain that their practical application is not always easy. Moreover, in localizing a diastolic murmur, we have not the advantage of those rules as regards difference of situation in which the maximum of intensity is observed, and the different directions in which the murmur is propagated, which are so useful in discriminating between mitral and aortic *systolic* murmurs.

The normal intensity of aortic second sound, or otherwise, divests this problem in diagnosis of much of its difficulty. If the aortic sound preserve its normal intensity, this is evidence against the aortic origin of the murmur, and of course, in favor of its being referable to the mitral orifice. Abnormal weakness of the aortic sound, on the other hand, is evidence that the murmur is not mitral, but referable to the aorta. I need not add that in resolving this problem, other circumstances are to be considered. My object has been simply to point out the importance of the aortic second sound in this connection.

I have endeavored in this letter to present, with great brevity, the conclusions drawn from the clinical study of the *second* or *diastolic* sound of the heart in health, together with the abnormal modfications of this sound, which are of most importance in their diagnostic bearings. The latter, not less than the former, are based on clinical studies, and were it consistent with the limitsof this communication, I could have adduced cases illustrative of the practical points which have been noticed.* I am well aware that to those readers who have given little or no attention to physical exploratoin as applied to the diagnosis of cardiac affections, these points will seem obscure. This is unavoidable, and not to be regretted should any be led thereby to cultivate a field of practical medicine not less attractive than important. In a subsequent letter I propose to consider the clinical study of the *first* or *systolic* sound of the heart in health and disease. In the mean time, I remain, with much respect, yours,

A. F.

* The reader will find illustrative cases by the writer in the Transactions of the American Medical Association for the present year.

